# Sociotechnical-systems analysis of IoT-AI convergence in cosmetic health

**DOI:** 10.3389/fmed.2026.1677802

**Published:** 2026-03-20

**Authors:** Abdulrahman Makhseed, Husain Arian, Ali Shuaib

**Affiliations:** 1 Department of Plastic Surgery, Jaber Al-Ahmad Hospital, Ministry of Health, Kuwait City, Kuwait; 2 Department of Plastic Surgery, Jahra Hospital, Ministry of Health, Al Jahra, Kuwait; 3 Biomedical Engineering Unit, Department of Physiology, College of Medicine, Kuwait University, Kuwait City, Kuwait

**Keywords:** artificial intelligence (AI), cosmetic health, digital health, Internet of Things (IoT), personalized beauty, preventive healthcare, sociotechnical systems

## Abstract

The convergence of Internet of Things (IoT) devices and artificial intelligence (AI) in cosmetic health offers significant potential for preventive healthcare and personalized beauty-health integration. Despite market growth, implementation of IoT-AI technologies remains fragmented due to misalignment between technical capabilities and social systems. This perspective article uses sociotechnical-system analysis to examine implementation challenges in digital beauty-health initiatives. The analysis revealed that devices prioritized technical accuracy over integration with user routines, applications achieved consumer adoption while creating workflow challenges for healthcare systems, and algorithms exhibited performance disparities across populations. Studies on sociotechnical systems in healthcare demonstrate that successful implementation requires joint optimization across technical infrastructure, social systems, organizational contexts, and environmental factors. We propose establishing relevant sociotechnical standards, validating integration through diverse trials, and achieving healthcare alignment to this end. The priority areas include UV monitoring, skin barrier assessment, and AI-driven personalization. Without coordinated action addressing accuracy, workflow integration, and algorithmic fairness, cosmetic IoT-AI risks amplifying existing disparities rather than democratizing personalized cosmetic health.

## 1 Introduction

The convergence of the global beauty industry with healthcare technology opens significant opportunity in preventive dermatology. Daily skincare routines engage billions of individuals worldwide, creating natural touchpoints for cutaneous health monitoring and early intervention (1). The global cosmetics market reflects substantial consumer engagement in personal care practices that, with appropriate technological infrastructure, could serve dual purposes (2).

Recent developments have produced relevant capabilities. These include handheld Internet of Things (IoT) enabled devices for measuring cutaneous parameters (3), artificial intelligence (AI) algorithms for personalized skincare recommendations (4–8), and smart mirrors capable of real time skin health assessment (9). Wearable sensor technologies have demonstrated potential for continuous dermatological monitoring (10–12). Despite this growing technical repertoire, attempts to integrate IoT and AI technologies into aesthetic health applications have encountered persistent adoption barriers.

The nature of these barriers is instructive. Diagnostic devices achieve high accuracy but offer limited practical usability. Smartphone applications democratize access but generate workflow challenges for healthcare systems unprepared to absorb consumer driven demand. Machine learning systems demonstrate technical sophistication but exhibit algorithmic bias across populations (12). Research on algorithm performance in dermatology has documented disparities of particular concern, with accuracy degradation up to 34% for darker phototypes (13). Systematic reviews have corroborated this finding across diverse patient populations (14, 15). These patterns cannot be explained by technical inadequacy alone. They demand examination of complex interplay between technology, human behavior, and systemic factors.

This perspective applies sociotechnical systems (STS) theory to analyze opportunities and challenges presented by cosmetic IoT and machine learning systems in health contexts. STS theory holds that optimal outcomes emerge from joint optimization of technical and social systems, rather than from maximizing either component in isolation (16, 17). Work on sociotechnical frameworks in healthcare has underscored the importance of considering technology, processes, and human factors as an integrated whole (18–20). Achieving rapid learning health systems requires sociotechnical harmonization between research and operational IT infrastructure (21). Our central argument is that cosmetic IoT and machine learning systems will enhance health only through deliberate optimization of sociotechnical interfaces where aesthetic routines meet health monitoring, consumer devices encounter clinical workflows, and individual wellness intersects with population health systems.

## 2 The sociotechnical framework for cosmetic health technology

The technical infrastructure encompasses hardware elements including miniaturized IoT sensors for measuring cutaneous parameters (22), connectivity modules enabling data transmission, and edge processors performing local analysis. Software components include machine learning algorithms for image analysis, data processing pipelines for managing sensor streams, and user interfaces for presenting health insights. Recent work has demonstrated feasibility of transparent ultraviolet (UV) photodetectors for real time wearable photoprotection monitoring (11) alongside flexible passive wireless sensing platforms for multimodal health assessment (10). Critical considerations include epidermal barrier function measurement (23) and performance metrics spanning both aesthetic outcomes and health indicators. These integration challenges mirror broader issues documented for machine learning medical devices (24) and IoT healthcare applications more generally (25).

Understanding the implementation of IoT-AI in cosmetics requires analyzing the interconnected subsystems that determine the success or failure of technology implementation. Building on established STS approaches in healthcare (16, 17, 26), we identified four critical subsystems, namely, technical infrastructure, social systems, organizational context, and environmental subsystems, whose alignment shapes outcomes in cosmetic health applications.

The social system comprises human networks that interact with IoT-AI technology for cosmetics. Primary users, individuals performing daily beauty routines, exhibit established behaviors, aesthetic preferences, and varying levels of health consciousness. Stakeholders include beauty professionals who may integrate devices into their services, healthcare providers who could receive health alerts, and social networks that influence beauty practices. This subsystem operates through deeply ingrained behavioral patterns, requiring technology to complement rather than disrupt existing habits.

The organizational context defines the structural boundaries within which sociotechnical systems operate. Beauty industry factors include retail channels, professional services, and product development cycles. Healthcare organizational factors include dermatology practices, primary care integration pathways, and insurance reimbursement structures. Regulatory frameworks encompass cosmetic regulations and medical device requirements when health claims are made.

The environmental subsystem includes broader societal factors that shape adoption. Cultural beauty standards vary dramatically across regions, influencing the parameters that users monitor and interpret. Socioeconomic disparities affect access to beauty products and health technology. Privacy expectations regarding biometric data differ between cultures and generations.

**FIGURE 1:**
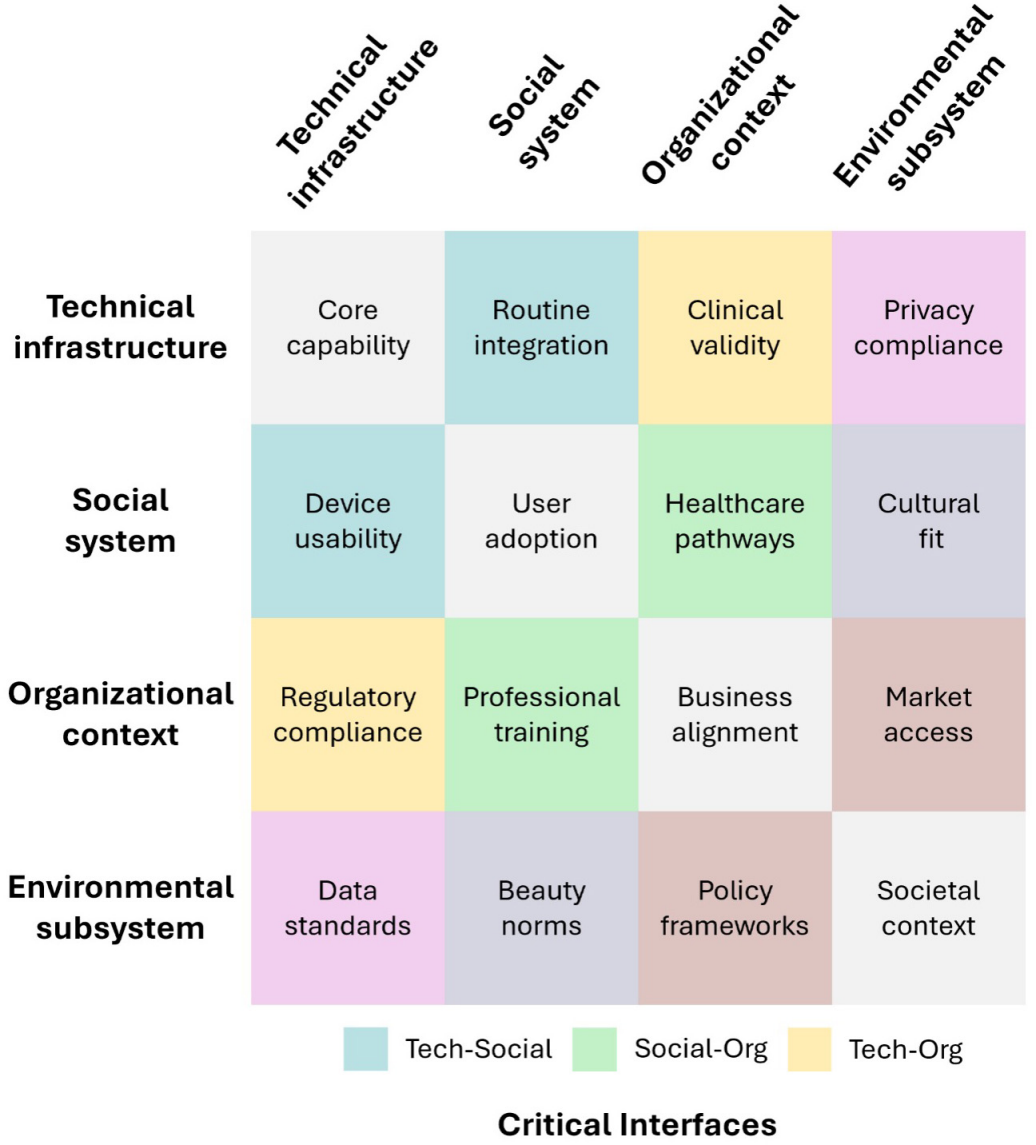
Sociotechnical interaction matrix for IoT in cosmetics. The matrix illustrates the bidirectional relationships between four critical subsystems: technical infrastructure, social system, organizational context, and environmental subsystem.

Based on the core principle of STS theory that system properties emerge from subsystem interactions rather than component excellence (16), successful implementation requires optimizing interfaces where subsystems meet, creating synergistic rather than conflicting interactions (Figure 1).

Figure 1 shows bidirectional subsystem interactions, with intersections representing alignment or conflict sites. The Technical Infrastructure and Social System interface determines device adoption. Capabilities aligned with existing skincare behaviors enable integration, whereas imposed functionalities create barriers. The Organizational Context and Environmental Subsystem interface shows regulatory adaptation to evolving beauty standards and privacy expectations. The Social System and Organizational Context intersection reveals how consumer-initiated dermatology referrals from cosmetic AI alerts overwhelm clinical capacity. The Environmental Subsystem and Technical Infrastructure connection demonstrates algorithmic bias from skin tone disparities in training data. Interface analysis identifies misalignment sites, prioritizing system optimization over component performance in sociotechnical versus technology-centric implementation.

## 3 Analysis of implementation patterns and opportunities

### 3.1 Patterns of sociotechnical misalignment

High-end skin analysis systems using multispectral imaging and advanced algorithms characterize skin with remarkable precision yet face adoption barriers. These systems require users to travel to specific locations, undergo lengthy measurement procedures, and interpret clinically dense reports (3, 9). Greenhalgh et al. demonstrated that technical excellence decoupled from usability and workflow fit reliably predicts non-adoption and abandonment (26). The NASSS framework identifies mismatch between technology complexity and adopter capacity as the root cause. Cosmetic devices fit this model. Diagnostic accuracy cannot overcome fundamentally social and behavioral barriers.

The disconnect between measurement capability and clinical utility appears prominently in epidermal barrier monitoring. Barrier function biology is well established (23), and standardized transepidermal water loss (TEWL) measurement guidelines provide robust clinical frameworks (27). However, consumer devices rarely achieve measurement consistency across repeated daily use needed to track meaningful barrier integrity trends. Sensors performing well under controlled laboratory conditions produce noisy, unreliable data when used in home environments at varying temperatures and humidity. Barrier function interpretation requires dermatological expertise most consumers lack. Algorithmic sophistication cannot bridge this gap.

Smartphone based dermatological screening applications present the opposite problem. These tools optimize accessibility while ignoring systemic consequences. Applications downloaded by millions achieve adequate technical performance and align with existing phone usage patterns. Problems emerge when algorithms flag suspicious lesions or inflammatory conditions. Anxious users seek dermatology appointments, but healthcare systems are designed around symptomatic presentation, not consumer generated alerts. Udrea et al. demonstrated that machine learning algorithms for lesion triage perform reasonably under controlled conditions, but false positive rates in real world consumer use generate unanticipated clinical burden (28). Consumer success becomes system level disruption. Broader evidence on direct to consumer health technologies confirms this as a recurring pattern when clinical validation pathways are inadequately established (29).

Beauty applications powered by machine learning expose a third failure mode rooted in societal development context. Training datasets skewed toward lighter skin phototypes and Western aesthetic ideals produce algorithms with measurable performance gaps across Fitzpatrick skin types. Daneshjou et al. reported accuracy degradation up to 34% for darker phototypes on diverse clinical image sets (13). Systematic reviews independently corroborated this across acne vulgaris diagnosis (14) and sexually transmitted infection identification (15). Applications marketed as democratizing aesthetic confidence may reinforce discriminatory standards they claim to transcend. This mirrors broader algorithmic bias in healthcare, where training data inequities propagate through downstream applications (30).

### 3.2 Current prospects for sociotechnical alignment

Recent developments suggest opportunities for successful implementation through genuine socio-technical integration. Innovations increasingly address sociotechnical requirements rather than pursuing isolated excellence. Research has demonstrated the feasibility of handheld IoT devices for achieving seamless beauty routine integration, rapidly measuring multiple skin parameters with cloud-based analysis without disrupting the user experience (3). Smart mirror technologies involve embedding sensors in existing bathroom fixtures for skin analysis during natural grooming, with AI tracking changes over time (9).

Wearable UV sensors have successfully demonstrated form-factor innovations aligned with beauty practices. Recent advances in transparent UV photodetectors have enabled continuous monitoring while maintaining optical transparency (11), which is essential for integration into wearable and transparent electronic devices. Studies evaluating UV-sensor wearable devices in melanoma survivors have provided insights into the real-world implementation challenges and user engagement patterns (31). Advanced flexible sensors achieve medical-grade measurements while maintaining cosmetic acceptability (32). These developments reflect broader advances in wearable sensor technologies (33) and multimodal sensing platforms (10).

The skincare as self-care movement encourages the adoption of IoT-AI technologies by elevating beauty routines from vanity to wellness practices. Social media normalizes detailed skin analysis, whereas quantified self-trends naturally extend to beauty metrics. Professional beauty services increasingly incorporate health elements, creating venues that bridge aesthetic treatment and health monitoring. The integration challenges documented in healthcare applications (29) provide lessons for the beauty-health convergence.

Growing awareness of skin cancer has made sun protection a mainstream beauty concern, while clean beauty movements have demanded transparency about the health impacts of products. Cultural beauty standards show increasing diversity and health consciousness as consumers seek evidence-based solutions to their problems. Privacy frameworks have evolved to address beauty and health data concerns, creating regulatory structures that enable secure data sharing while protecting user privacy. These converging trends create opportunities for IoT-AI technologies to genuinely serve both beauty and health objectives.

## 4 Implementation strategy

### 4.1 Phased sociotechnical optimization

**FIGURE 2:**
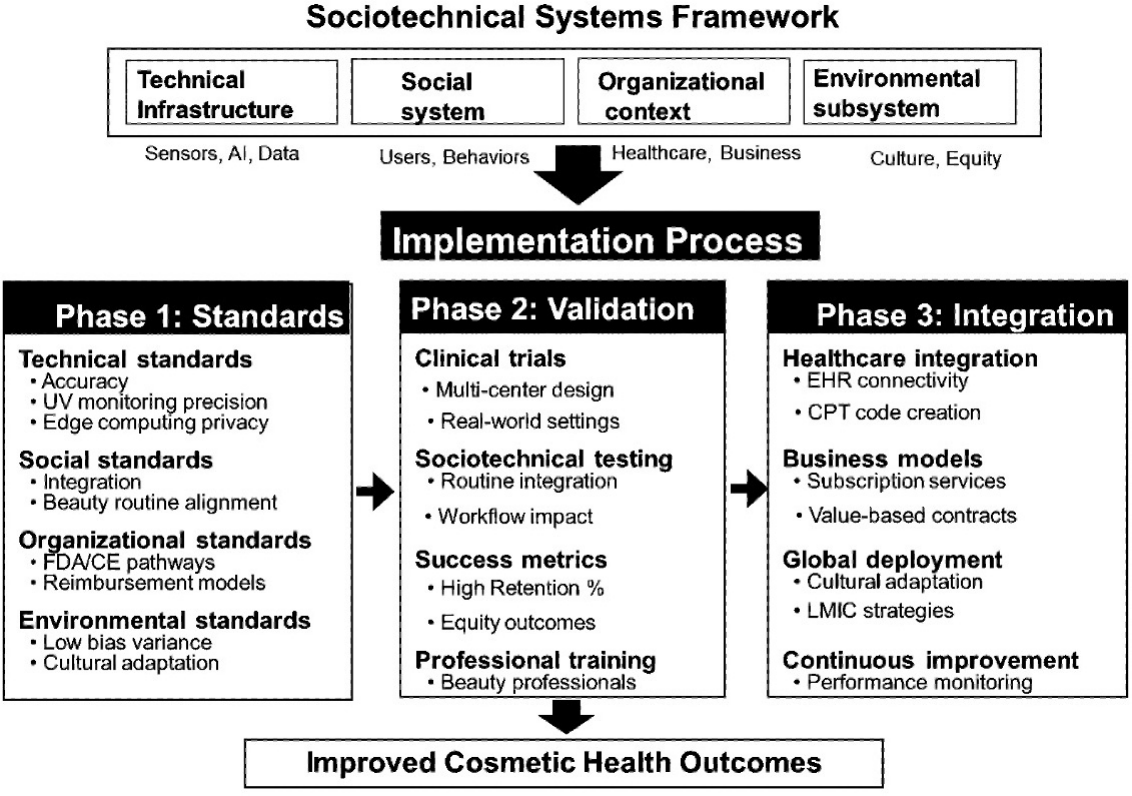
Three-phase implementation pathway. Phase 1 establishes standards across subsystems, Phase 2 validates integration through real-world trials, and Phase 3 enables scaling through interoperability and sustainable business models.

Successful implementation requires coordinated optimization across all subsystems using a three-phase approach (Figure 2). Phase 1 established technical standards specifying minimum performance thresholds for health-relevant measurements while accommodating beauty application constraints. Standards development should consider the U.S. Food and Drug Administration (FDA) guidelines for software as a medical device and the European Union (EU) Medical Device Regulation. For UV monitoring, an accuracy within ±10% of laboratory instruments may suffice for photoaging prevention guidance. Skin hydration measurements require consistency across daily use rather than absolute accuracy, enabling trend tracking for barrier function monitoring based on established guidelines for transepidermal water loss measurements (27). Social acceptance standards require participatory development involving beauty professionals, dermatologists, and diverse consumer groups. Organizational standards must clarify these regulatory pathways. Environmental equity standards require explicit attention to performance variation across populations to address algorithmic bias concerns documented in recent systematic reviews (14, 15, 30).

Phase 2 prioritizes clinical validation through testing complete sociotechnical systems in natural use contexts rather than controlled settings, following established reporting standards for diagnostic accuracy studies (34) and implementation science frameworks (35). Trial designs should evaluate user engagement maintenance, health insight effects on behavior, and adoption success across diverse populations. Multisite trials must represent varied beauty cultures and healthcare systems, with validation metrics extending beyond clinical accuracy to sociotechnical outcomes, including sustained engagement rates and appropriate help-seeking behavior. Research on retention in digital health studies (36) provides guidance for measuring sustained engagement.

Phase 3 focuses on scaling the implementation, which requires simultaneous optimization across subsystem interfaces. Technical interoperability must enable data flow between beauty applications, professional records, and healthcare systems while respecting privacy preferences through advanced privacy-preserving techniques (37, 38). Regulatory frameworks governing beauty-health data create complex compliance requirements (39–41). In the European Union, biometric data from IoT cosmetic devices constitute special category data under Article 9 of the General Data Protection Regulation (GDPR), requiring explicit consent and enhanced security measures. In the United States, when beauty-health data interfaces with clinical systems or generates health alerts for healthcare providers, it may trigger Health Insurance Portability and Accountability Act (HIPAA) compliance requirements for protected health information. These regulatory mandates necessitate robust consent mechanisms, data minimization principles, and clear user controls over data sharing between cosmetic and healthcare contexts. Security considerations include emerging approaches to ensure health data integrity and secure computation (42, 43). Business model innovation should align with diverse stakeholder incentives through subscription models, brand partnerships, and healthcare system collaborations. Professional development programs must prepare beauty and healthcare workers for IoT-AI convergent practice.

Implementation choices determine whether technologies reduce or amplify existing disparities in beauty and healthcare access. High-income countries risk creating two-tier systems in which advanced technologies remain accessible only to affluent consumers. Preventing this requires integration with mass-market retailers and community health centers. Low- and middle-income countries have opportunities to address the limited availability of dermatologists through AI-guided beauty professionals; this requires algorithms trained on local populations and business models adapted to local distribution channels. Cultural adaptation extends beyond translation to the fundamental redesign of beauty-health integration. Regions with traditional beauty practices require complementary technologies to work alongside existing practices rather than replacing them. Communities exhibiting medical mistrust require trusted beauty professionals to serve as intermediaries between consumers and healthcare systems.

## 5 Discussion

This sociotechnical analysis shows that the cosmetic IoT-AI convergence represents an inflection point for preventive healthcare democratization, aligned with broader digital health trends. The convergence of technical capabilities, social acceptance, and environmental factors can create unprecedented opportunities for transforming routine beauty practices into health-monitoring behaviors. Previous implementation failures provide essential guidance extending beyond cosmetic applications to wider digital health challenges (29, 44). Technical excellence without user integration creates barriers, regardless of capability, a pattern observed across numerous healthcare technologies (26). Consumer adoption without system preparation creates chaos, as evidenced by challenges with smartphone applications, paralleling broader issues with direct-to-consumer health technologies. Algorithmic development without environmental consideration perpetuates harm, reflecting systemic challenges in AI fairness across healthcare applications (30). These lessons emphasize that success requires collaborative optimization across traditionally separate domains, consistent with the established sociotechnical principles (15).

Current opportunities demonstrate that sociotechnical alignment is achievable when development prioritizes integration. Technical innovations increasingly reflect lessons learned from broader digital health implementations, prioritizing integration over isolated performance. The success of recent wearable UV sensors (11, 31) and flexible sensing platforms (10, 31) shows how form-factor innovation aligned with existing behaviors can overcome adoption barriers. Social systems are embracing beauty-health convergence through wellness movements that match the expansion of quantified-self trends into diverse health domains. The professional evolution in beauty services mirrors broader healthcare digitization trends, where traditional sectoral boundaries are becoming increasingly permeable. Environmental factors supporting integration reflect broader societal shifts toward health-conscious consumption and data transparency. However, capturing these opportunities requires coordinated action across stakeholders to address the coordination challenges that have hindered previous digital health initiatives (25, 29).

Most importantly, this analysis shows the potential of cosmetic IoT-AI for amplifying either global health equity or disparity, echoing broader concerns about digital health technologies (30). The democratization potential stems from the universal engagement patterns of beauty, in contrast to the episodic nature of healthcare treatments. However, the risk of perpetuating or amplifying existing disparities is substantial, as demonstrated by the current AI bias in cosmetic applications (13–15). Choices made during the current development will determine whether these technologies democratize personalized health monitoring or create new forms of the digital divide.

This study has several limitations. Although the framework is inclusive, it may not capture all relevant factors in the rapidly evolving cosmetic and healthcare landscapes. Moreover, the analysis relied primarily on existing examples and theoretical frameworks, and the proposed implementation roadmap requires empirical validation. Additionally, cultural variations in beauty practices and healthcare systems may necessitate more tailored approaches than those suggested by the general framework. Future research should develop specific metrics for sociotechnical optimization to guide implementation decisions and evaluation criteria. The empirical validation of the proposed phases through pilot implementation across diverse populations and settings is essential. Economic evaluation frameworks must be adapted to assess beauty-health convergence value proposals for multiple stakeholders. The regulatory landscape also requires continued attention as agencies worldwide grapple with beauty and health boundary definitions.

## 6 Conclusion

The opportunity to transform daily beauty practices into health-monitoring touchpoints represents one of the most significant preventive healthcare opportunities of the digital age. However, realizing this potential demands immediate and coordinated attention from technologists, healthcare providers, beauty industry leaders, regulators, and researchers alike. The sociotechnical framework presented here provides a foundation for this effort; however, success will depend on a sustained commitment to optimization across all system dimensions. The convergence of beauty and health through IoT-AI represents more than just technological advancement. Achieving this convergence requires abandoning siloed thinking in favor of sociotechnical optimization that serves all populations.

## Data Availability

The original contributions presented in this study are included in the article/supplementary material, further inquiries can be directed to the corresponding author.
